# New indicators and indexes for benchmarking university–industry–government innovation in medical and life science clusters: results from the European FP7 Regions of Knowledge HealthTIES project

**DOI:** 10.1186/s12961-019-0414-5

**Published:** 2019-01-28

**Authors:** Laurel D. Edmunds, Silvia Gluderer, Pavel V. Ovseiko, Roel Kamerling, Jacqueline Ton, Laura Vis, Mario Jenni, Gregory Tutton, Helen Lawton-Smith, Márta Völgyiné Nadabán, Máté Rab, Jon Rees, John Anson, Alexander D. Rushforth, Maxine Allen, Alastair M. Buchan, Montserrat Vendrell, Alex Casta, Gábor Mehes, Pancras C. W. Hogendoorn, Ernst Hafen, A. Bassim Hassan

**Affiliations:** 1 Sir William Dunn School, University of Oxford, Oxford, United Kingdom; 2 Astellas Pharma, Zürich, Switzerland; 3 Medical Delta, Delft University of Technology, Delft, The Netherlands; 4 Leiden University Medical Center, Leiden, The Netherlands; 5 Province of South Holland, The Hague, The Netherlands; 6 Bio-Technopark Schlieren-Zürich, Zurich,, Switzerland; 7 Cloverton Holdings, Basel, Switzerland; 8 Department of Management, Birkbeck, University of London, London, United Kingdom; 9 Independent Consultant, Debrecen, Hungary; 10 Innonic Group, Debrecen, Hungary; 11 Jon Rees Associates Ltd, Oxford, United Kingdom; 12 Oxford Gene Technology, Oxford, United Kingdom; 13 Nuffield Department of Primary Care Health Sciences, University of Oxford, Oxford, United Kingdom; 14 Business Development Team, Medical Sciences Division, University of Oxford, Oxford, United Kingdom; 15 BioCat, Bioregion of Catalonia, Barcelona, Spain; 16 Faculty of Public Health, University of Debrecen, Debrecen, Hungary; 17 Institute of Molecular Systems Biology, ETH Zürich, Zurich, Switzerland

**Keywords:** Regional innovation cluster, innovation index, triple helix, university–industry–government innovation, Regions of Knowledge, life sciences, medical sciences, biotechnology, public policy, European Union

## Abstract

**Background:**

While the European Union is striving to become the ‘Innovation Union’, there remains a lack of quantifiable indicators to compare and benchmark regional innovation clusters. To address this issue, a HealthTIES (Healthcare, Technology and Innovation for Economic Success) consortium was funded by the European Union’s Regions of Knowledge initiative, research and innovation funding programme FP7. HealthTIES examined whether the health technology innovation cycle was functioning differently in five European regional innovation clusters and proposed regional and joint actions to improve their performance. The clusters included BioCat (Barcelona, Catalonia, Spain), Medical Delta (Leiden, Rotterdam and Delft, South Holland, Netherlands), Oxford and Thames Valley (United Kingdom), Life Science Zürich (Switzerland), and Innova Észak-Alföld (Debrecen, Hungary).

**Methods:**

Appreciation of the ‘triple helix’ of university–industry–government innovation provided the impetus for the development of two quantifiable innovation indexes and related indicators. The HealthTIES H-index is calculated for disease and technology platforms based on the h-index proposed by Hirsch. The HealthTIES Innovation Index is calculated for regions based on 32 relevant quantitative and discriminative indicators grouped into 12 categories and 3 innovation phases, namely ‘Input’ (*n* = 12), ‘Innovation System’ (*n* = 9) and ‘Output’ (*n* = 11).

**Results:**

The HealthTIES regions had developed relatively similar disease and technology platform profiles, yet with distinctive strengths and weaknesses. The regional profiles of the innovation cycle in each of the three phases were surprisingly divergent. Comparative assessments based on the indicators and indexes helped identify and share best practice and inform regional and joint action plans to strengthen the competitiveness of the HealthTIES regions.

**Conclusion:**

The HealthTIES indicators and indexes provide useful practical tools for the measurement and benchmarking of university–industry–government innovation in European medical and life science clusters. They are validated internally within the HealthTIES consortium and appear to have a degree of external prima facie validity. Potentially, the tools and accompanying analyses can be used beyond the HealthTIES consortium to inform other regional governments, researchers and, possibly, large companies searching for their next location, analyse and benchmark ‘triple helix’ dynamics within their own networks over time, and to develop integrated public–private and cross-regional research and innovation strategies in Europe and beyond.

**Electronic supplementary material:**

The online version of this article (10.1186/s12961-019-0414-5) contains supplementary material, which is available to authorized users.

## Background

As policy-makers see innovation as a key driver of economic growth and wealth creation, the European Union (EU) is striving to become the ‘Innovation Union’ [1]. Despite substantial policy focus and investment in research and innovation at the regional level, comparing and benchmarking European regional innovation clusters is still in its formative stage and remains limited in scope. Benchmarking usually takes the form of observational studies of national innovation systems, sometimes with extended regional scoreboards. For example, the Regional Innovation Scoreboard was developed as an extension of the Innovation Union Scoreboard to measure the innovation performance of European regions on a limited number of indicators [2–4]. The Regional Ecosystem Scoreboard places emphasis on the dynamics and conditions that characterise the quality and nature of innovation and entrepreneurship in administrative regions, defined according to the Classification of Territorial Units for Statistics (NUTS) nomenclature of territorial units for statistical purposes [5]. The Cluster Observatory provides a range of macro- and micro-economic mapping data, reports on clusters and regional competitiveness conditions, information about cluster organisations, as well as educational resources [6]. In addition, a number of EU commissioned reports highlight the varied quantification of the different phases of the innovation cycle, and the identification of critical factors that stimulate clustering from both a ‘bottom-up’ and ‘top-down’ perspective [7, 8]. The primary objective of such scorecards and reports are the relative ranking of countries and large administratively defined regions, but they do not necessarily capture the workings and competitive advantages of actual innovation clusters from a regional co-ordination perspective [9, 10]. Moreover, it remains unlikely that they are sensitive to variations between industry sectors, and therefore objective and quantifiable data tailored to the biotech, medical technology and pharma sectors are required for effective benchmarking and time dependent analysis [7, 11].

Herein, we focus on actual regional innovation clusters, defined as a critical mass of research innovation in academic institutions and companies that are located within a relatively small geographic area [12, 13]. Geographic clustering allows companies to derive competitive advantage, not only from their own resources and capabilities, but also from the shared resources and capabilities located in their geographical proximity [14]. Such geographic clustering is considered to encourage cross-sector networks, spin-out companies, investment, collaborations and regional development, particularly when there is a strong presence of innovative firms from the same industry [15]. Interactions between large companies, small- to medium-sized enterprises and research organisations that lead to the sharing and exchange of facilities, knowledge and people, are likely to contribute to technology transfer. Importantly, these interactions may be catalysed through extended networking, personnel and information dissemination within and between clusters [9]. Regions that have developed such clusters, especially those with a strong presence of universities and knowledge-based industry, are considered to have enhanced economic competitiveness, innovation and growth [16]. Given the benefits of clustering, national governments, the European Commission and intergovernmental organisations such as the OECD, advocate creating regional clusters, particularly in the areas that maximise public good [16–21]. The ‘triple helix’ of university–industry–government relations to increase the potential for innovation and wealth creation is now developed through new organisations such as science parks and incubators that cut across the university–industry–government boundaries. Thus, quantitative benchmarking of regional clusters is an essential prerequisite for devising strategies and taking actions to accelerate the impact of regional medical and life science clusters on health and wealth creation.

In the field of science, technology and innovation (STI) studies, there are six established sources of data and related approaches [22] that can be potentially used to quantify and measure innovation with a high degree of comparability across a number of diverse regions. First, national-level research and development (R&D) data are used to characterise national contexts and inputs into the innovation process as well as innovation activities [23]. Second, patents and citations provide insights into the invention process [24–27]. Third, bibliometrics help understand and forecast the scientific process underpinning inventions [28, 29]. Fourth, technometrics rely on expert opinion to assess technological change and its policy implications [30, 31]. Fifth, topic-specific databases and innovation surveys provide statistics on collaboration, commercialisation, financing and other innovation activities and opportunities [32–34]. Finally, composite synthetic indicators use a variety of data sources to assess innovation capabilities and performance [35, 36].

Building on the established STI approaches, we report and discuss methods and results pertaining to the development of new HealthTIES (Healthcare, Technology and Innovation for Economic Success) indicators and indexes for benchmarking university–industry–government innovation in European medical and life science clusters. The HealthTIES project was part of the €126 million (2007–2013) Regions of Knowledge initiative supported by the European Commission under the research and innovation funding programme FP7 [37]. The aim of the initiative was “*to support trans-national mutual learning and cooperation between research-driven clusters, bringing together regional authorities and development agencies, public research organisations, industry and other relevant stakeholders*” [37]. The main activities developed by HealthTIES to achieve the aims of Regions of Knowledge were to complete a detailed set of quantitative assessments for the analysis of regional clusters, including the development of a ‘virtual reference cluster’, to develop a mentoring strategy between a highly developed cluster (Life Science Zürich) with one less developed (Debrecen, Hungary), and to develop a set of regional action plans to improve the integration of contributors to regional economies.

Given that the primary focus of our project was on the HealthTIES regions, we initially disseminated and used the results of the project to inform actions within the regions. Yet, there are a paucity of such research outputs from this and other projects in the public domain and peer-reviewed literature [38], similar to what is observed for other European-funded projects and initiatives. A recent study in *Health Research Policy and Systems* demonstrated that, although significant public resources are invested in European-funded health projects, data and reports from such projects are often unavailable or inaccessible [39]. Globally, billions of dollars in research investment are wasted when full information about health research studies is inaccessible [40]. In line with the objectives of Responsible Research and Innovation [41], which increases the value of European research investment, we herein make the results from the HealthTIES project publicly available in Open Access format.

## Methods

### Aim and objectives

We established the HealthTIES consortium [42] to deliver on the stated aim of the Regions of Knowledge by examining whether the health technology innovation cycle was functioning differently in five European regional innovation clusters, and to propose regional and joint actions to improve their performance. The specific objectives were as follows:

- develop indicators and indexes for assessing knowledge and innovation capital and the functioning of the innovation cycle;
- identify key stakeholders and collect data to operationalise the proposed indicators and indexes;
- propose joint and regional actions to improve regional performance through benchmarking and mutual learning.

### Study setting

**Fig. 1:**
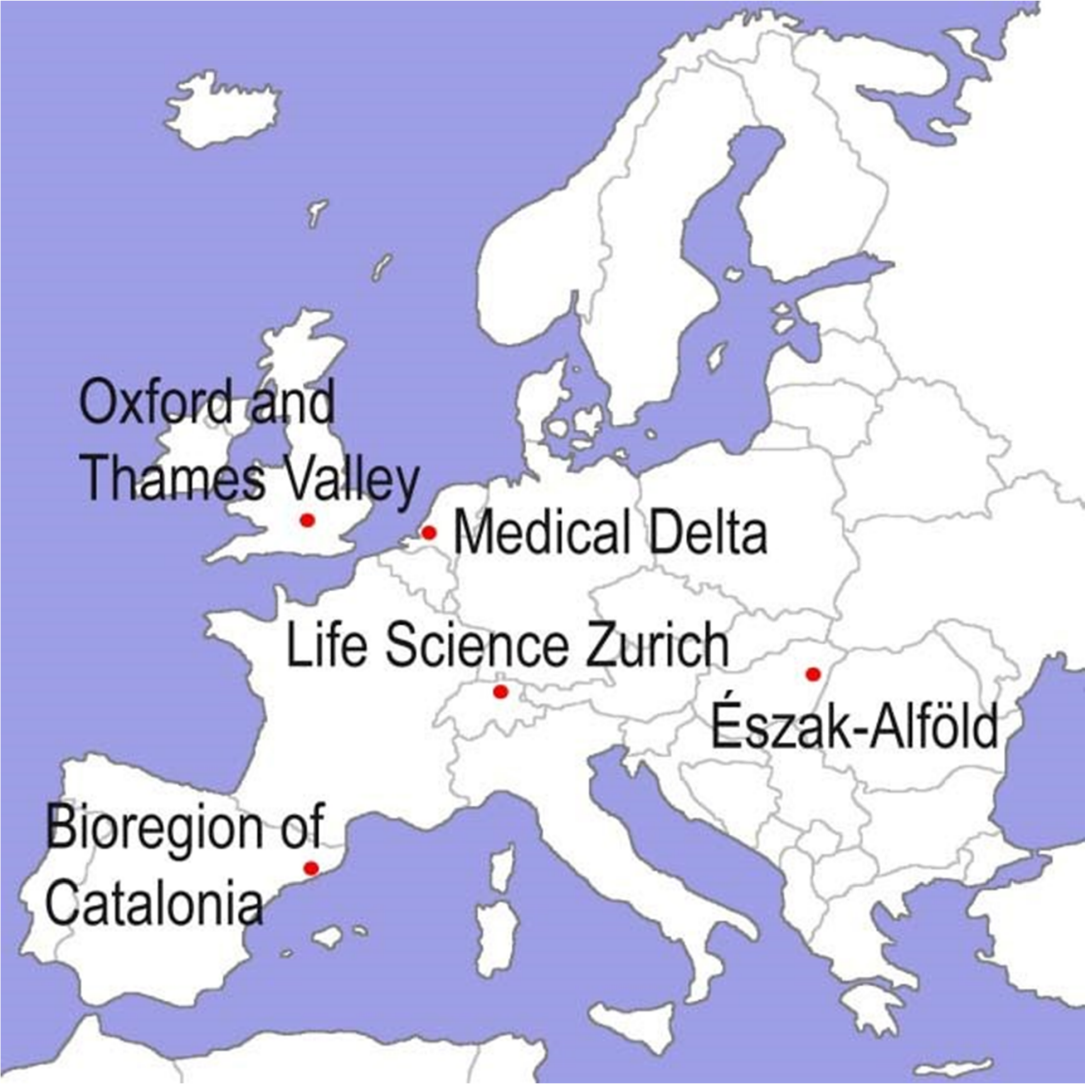
The HealthTIES (Healthcare, Technology and Innovation for Economic Success) consortium

The HealthTIES consortium consisted of five European regional clusters centred around the following cities (Fig. 1):

- Barcelona (Bioregion of Catalonia, Catalonia),
- Leiden, Rotterdam, and Delft (Medical Delta, South Holland),
- Oxford (Oxford and Thames Valley, United Kingdom),
- Zürich (Life Science Zürich, Switzerland),
- Debrecen (Innova Észak-Alföld, Hungary).

We selected the first four participating clusters on the basis of pre-existing bilateral collaboration between cluster members and their proven track record in medical and life sciences research and innovation [38]. Innova Észak-Alföld was selected as a mentoring cluster on the basis of its potential to accelerate research-driven innovation. Hungary has one of the most developed biotechnology sectors among the new EU member states, and Debrecen had attracted significant investments from Gedeon Richter Plc. to build a new biotechnology production facility that is unique in Central Eastern Europe [43].

### Theoretical framework

To guide data collection, analysis and actions, this study used the established ‘triple helix’ model of university–industry–government relations based on the theoretical insight that universities, industry and government are becoming increasingly interdependent and co-evolving, while retaining their institutional identities [17, 21, 44–46]. Imperative to the functioning of the ‘triple helix’ is the creation of the ‘knowledge space’ to intensify research and generate new knowledge through collaboration, the ‘consensus space’ to build relationships, agree on objectives and develop joint actions, and the ‘innovation space’ to implement joint actions by bringing together knowledge, business expertise and venture capital. The ‘new organisational actors’ or ‘hybrid organisations’, such as science parks, incubators and new forms of venture capital, which span traditional university–industry–government boundaries, are shown to be effective in creating the aforementioned ‘spaces’ [21, 47, 48].

Under the assumptions of ‘triple helix’ model, maximising interactions, creating ‘spaces’ and new organisations that cut across the university–industry–government boundaries are proposed to increase the potential for innovation and wealth creation through free movement of people, knowledge and venture capital between universities, industry and government.

### Data collection and analysis

For each cluster, we collected and analysed the most recent data pertaining to universities, industry, government and new boundary-spanning organisations during the lifespan of the project (2010–2013). Most of our cross-sectional data and analyses refer to 2010 or 2012. We used a variety of well-established STI sources of data and approaches to data analysis. Namely, we collected and analysed R&D data from statistical offices and institutions, bibliometric data, public and commercial databases, and a number of readily available composite synthetic indicators. These data and analyses formed the basis of the HealthTIES H-index for disease and technology platforms and the HealthTIES Innovation index focusing on four disease fields (cardiovascular disease, cancer, neurodegenerative disease, immunology and infectious disease) and three technology platforms (molecular technology, imaging, and drug design, development and delivery).

We selected these disease fields and technology platforms on the basis of the concurrent assessment of the burden of mortality and morbidity in Europe as well as scientific and technological change and by the project’s experts as the most promising for improving the health of European citizens and making European health systems more sustainable in the context of the increasingly ageing population. Cardiovascular disease, cancer, infectious diseases and neurodegenerative diseases are the leading causes of mortality and morbidity in Europe. Frequently, they are manifestations of patient/gene–environment interactions. Therefore, interventions directed at environmental risk factors, such as alcohol, obesity and tobacco, are likely to have important but mixed benefits depending upon underlying genetic factors and age at exposure. Recent scientific and technological breakthroughs in molecular technology and imaging, as well as drug design, development and delivery have opened up new opportunities for primary prevention of these diseases through risk factor identification and secondary prevention through early and accurate diagnosis, as well as more appropriate and effective treatments.

### HealthTIES H-index for disease and technology platforms

To assess the knowledge and innovation capital in each region we used bibliometric h-indexes [49]. Although most commonly h-indexes are calculated for individual researchers, they could also be calculated for regions, diseases and technologies. We calculated h-indexes using the Clarivate Web of Science, formerly Web of Knowledge, research platform (www.webofknowledge.com) to benchmark our regions in four relevant disease fields and three technology platforms. Our protocol and search terms are shown in Additional file 1: Box S1 and Table S1, respectively. The HealthTIES h-index (HT H-index) for publications in a region in a specific field specifies the number *HT* of scientific articles for a given keyword (i.e. cardiovascular) for a given address (e.g. Zürich) that have been cited in other articles at least *HT* times according to Hirsch [49]. As the h-index depends on the length of a person’s scientific career, the HT H-index depends on the volume of research activity within clusters. The HT H-index permits the comparison of the relative strengths of a given location for specific keyword search terms and thus offers a valuable assessment of the research output of a cluster in a given disease or technology platform.

### HealthTIES innovation index

**Fig. 2:**
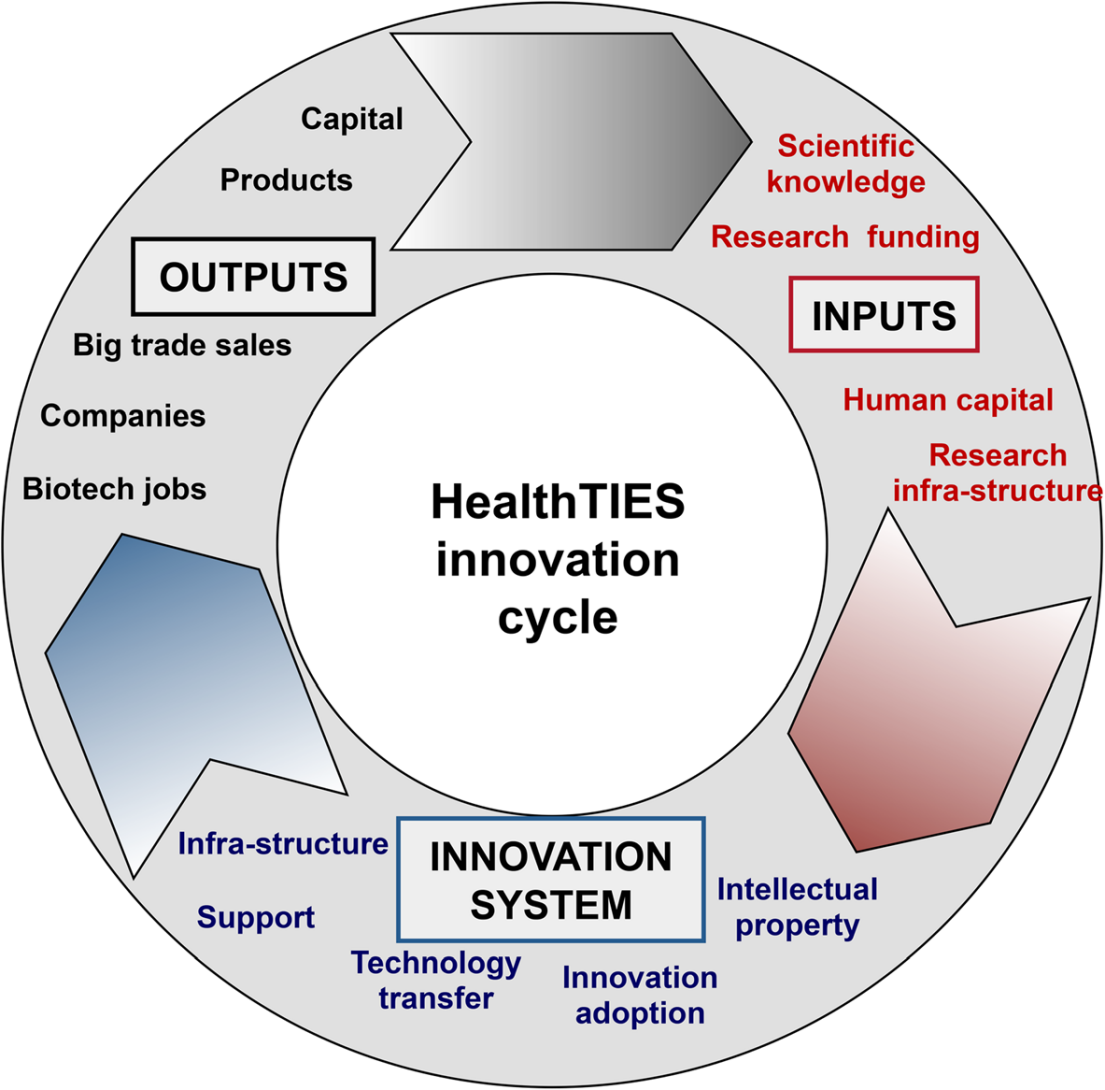
A logic model of the HealthTIES (Healthcare, Technology and Innovation for Economic Success) innovation cycle with key indicator categories

In an effort to create a ‘consensus space’ for building relationships, agreeing on objectives and developing joint actions under the assumptions of the ‘triple helix’ model, each cluster assembled a regional team, including representatives from small- to medium-sized enterprises, biotech parks, local government, hospitals, technology transfer offices (TTOs) and academia. Initially, we collected a wide range of data for each region, but it became clear that these data were either not necessarily available for each region, or were not comparable, or were not quantifiable. A workshop was convened to discuss the best data to collect and how to apply this to an index. All regional clusters were represented by 15 members, extending across every strand of the ‘triple helix’. We had discussions about the significance, validity, reliability and feasibility of the potential indicators facilitated by an independent consultant, and we reached consensus on which indicators to select. As a result, we developed a logic model of the innovation cycle consisting of three key phases, namely input, innovation system activity and outputs (Fig. 2).

We then compiled a list of potential indicators for each phase of the innovation cycle excluding those indicators for which no information was available or would not be feasible to collect. This process resulted in 32 quantitative indicators, which were grouped into 12 categories and three phases of the innovation cycle, namely ‘Input’ (*n* = 12), ‘Innovation System’ (*n* = 9) and ‘Output’ (*n* = 11) (Table 1). We also assigned, to each indicator, a weighting factor reflecting the relative significance of the underlying component in the innovation cycle. We determined mean scores for each weighting factor by voting among the 15 members present at the meeting, followed by wider circulation to all the members of the regional steering/advisory committees requesting their feedback, amendments and endorsement of the final set of indicators and weighting factors (Table 1).

**Table 1:** HealthTIES innovation indicators, data sources and weighting factors

Innovation phase	Category	Code	Indicator	General data source	Weighting factor
Input	Scientific knowledge	A01	Profs h-index > 30	University index of profs, Web of Science	18
		A02	Publications 2001–2010	Web of Science	16
	Research funding	A03	Research funding	Research institutions: annual reports, support staff	24
	Human capital	A04	International graduated MSc students	Research institutions: annual reports, support staff	3
		A05	International PhD students	Research institutions: annual reports, support staff	3
		A06	National graduated MSc students	Research institutions: annual reports, support staff	3
		A07	National PhD students	Research institutions: annual reports, support staff	3
		A08	Junior ERC grants 2007–2010	Lists of ERC-funded starting grants	8
		A09	Senior ERC grants 2008–2010	Lists of ERC-funded advanced grants	8
	Research infrastructure	A10	Research space m^2^	Research institutions: Facility managing staff	3
		A11	Research hospital beds	Websites and support staff of hospitals	3
		A12	Clinical trials phase I and II	ClinicalTrials.gov database	8
Innovation System	Intellectual property	B01	University spin-offs 2007–2010	TTO staff	17
		B02	Granted US patents 2007–2010	See-the-Forest Patent Analytics by IP Vision	14
	Innovation adoption	B03	WAIT indicator for countries	EFPIA Patients WAIT indicator	11
	Technology transfer	B04	University–industry research collaborations > €5 M	TTO/support staff of research institutions, EU project database Cordis	11
	Support	B05	TTO staff FTEs	TTO staff	13
		B06	National government procurement of advanced technology	WEF GCR 2010–2011, pillar 12.05	11
	Infrastructure	B07	National attractiveness (WEF index)	WEF GCR 2010–2011	12
		B08	Science parks m^2^	Science parks	6
		B09	Science park staff FTEs	Science parks	5
Output	Biotech jobs	C01	Jobs in biotech companies FTEs	Chambers of commerce, statistical offices	21.5
	Biotech companies	C02	Biotech companies < 20 FTEs	Chambers of commerce, statistical offices	5
		C03	Biotech companies > 20 FTEs	Chambers of commerce, statistical offices	10.5
	Big trade sales	C04	Big trade sales > €100 M 2001–10	Regional experts	5.5
	Products	C05	Products on market	BiotechGate database	11
		C06	Products in clinical trials	BiotechGate database	10
		C07	Products in discovery phase	BiotechGate database	5.5
		C08	Medicines available in countries	EFPIA Patient’s WAIT indicator	7
	Capital	C09	Total investments 2007–2010	BiotechGate database	8
		C10	Number of investments 2007–2010	BiotechGate database	8
		C11	Average Series A investments 2007–2010	BiotechGate database	8

*EFPIA* European Federation of Pharmaceutical Industries and Associations, *ERC* European Research Council, *FTE* full-time equivalent, *GCR* Global Competitiveness Report, *TTO* technology transfer office, *WAIT* Waiting to Access Innovative Therapies, *WEF* World Economic Forum

#### Input indicators

The final set includes 12 indicators to measure inputs in four categories. First, the numbers of highly cited professors and publications were deemed to be proxy indicators for the depth and breadth of the current scientific knowledge base. Given that the h-index reflects the duration of one’s scientific career, an h-index > 30 was chosen as a threshold for highly cited professors to also include younger researchers who had already made a significant impact on their respective fields. Second, the scope of the current research activity was measured through research funding. In doing so, currencies other than the euro were converted into euros using historical exchange rates. Third, both quantity and quality of human capital were considered to be central to current and future discoveries and innovation. We measured the numbers of graduated national and international MSc and PhD students as well as the numbers of European Research Council junior and advanced grants, which serve as a pan-European mark of research quality. Fourth, to approximate research infrastructure for current and future research, we measured research space, research hospital beds, and Phase I and II clinical trials. The latter indicate activity levels in proof of concept drug development.

#### Innovation system indicators

There are nine indicators to measure innovation system activity in three categories. First, the existing levels of innovation that were deemed to be reflected in intellectual property, university–industry technology transfer and adoption of innovation. Intellectual property and university–industry technology transfer were measured by the numbers of university spin-off companies, granted United States patents, and the number of university–industry collaborations larger than 5 million euros. The patients WAIT indicator (patients Waiting for Access to Innovative Treatment) was used to measure how fast innovations and new drugs are made available to the public [50]. Second, support for innovation was measured by the number of full-time equivalent (FTEs) posts in university TTOs in a given region and the extent of national government procurement of advanced technology. Third, the quality of innovation infrastructure was deemed to be reflected within the regional science park capacity and the overall national attractiveness. The capacity of regional science parks was measured by the size of space available in square meters and by the number of support staff in FTEs. The national attractiveness was deemed to be reflected by the World Economic Forum Global Competitiveness index [51].

#### Output indicators

There were 11 indicators to measure direct and more immediate outputs of the innovation system in five categories. First, the number of jobs in biotech companies was deemed to be one of the key indicators for economic growth and wealth creation. Second, we measured the number of non-subsidiary independent biotech companies, distinguishing between smaller companies (with fewer than 20 FTE employees) and larger companies (with 20 or more employees). Third, we measured the number of big trade sales (larger than 100 million euros), where small companies financed by venture capital or private equity were bought up by large life science, medical technology, biotech or pharma companies. Fourth, we measured the number of regional biotech companies’ products and the total number of medicines available nationally. The number of regional biotech companies’ products included those on the market, in clinical trials phases I–III and awaiting United States Food and Drug Administration approval, and in the pipeline, including discovery and lead optimisation. Finally, we measured capital as reflected in the total amount of investments (in euros), number of investments and the value of average Series A investments, i.e. the first significant venture capital investments. Given that there was no common publicly available data source for all of the HealthTIES regions, we operationalised products and capital indicators using the BiotechGate database (www.biotechgate.com) established by Venture Valuation Ltd., located in Zürich. BiotechGate is the largest life science company database in Europe and the United States, established primarily for the purposes of supporting partnering meetings. They allowed us to search our specific regions in their BiotechGate database (www.biotechgate.com). Although it is the most comprehensive database that is currently available, it is not exhaustive and is dependent on voluntary registration. Therefore, comparison of the indicators derived from this database may have known limitations.

### Comparison and benchmarking

All indicator data were converted into points, on linear scales ranging from 0 to 10, with 10 being the highest aspirational score. The highest actual data value of an indicator in any HealthTIES region was set at 7.5 points. This implied that each region may improve to 10 points in the future. This presentation method was applied to both HT Innovation and H-indexes. As stated, each HT Innovation indicator was then attributed by the HealthTIES consortium consensus, a relative weighting factor (Table 1). Overall scores were calculated by multiplying indicator points by the corresponding weighting factors. Category scores were obtained by adding up indicator scores within a category. Radar plots were used to visualise and compare indicator and category scores. The original benchmarking results for 2010 and 2012 are reported below and in Additional file 1.

## Results

### Comparative assessment of the knowledge and innovation capital

**Fig. 3:**
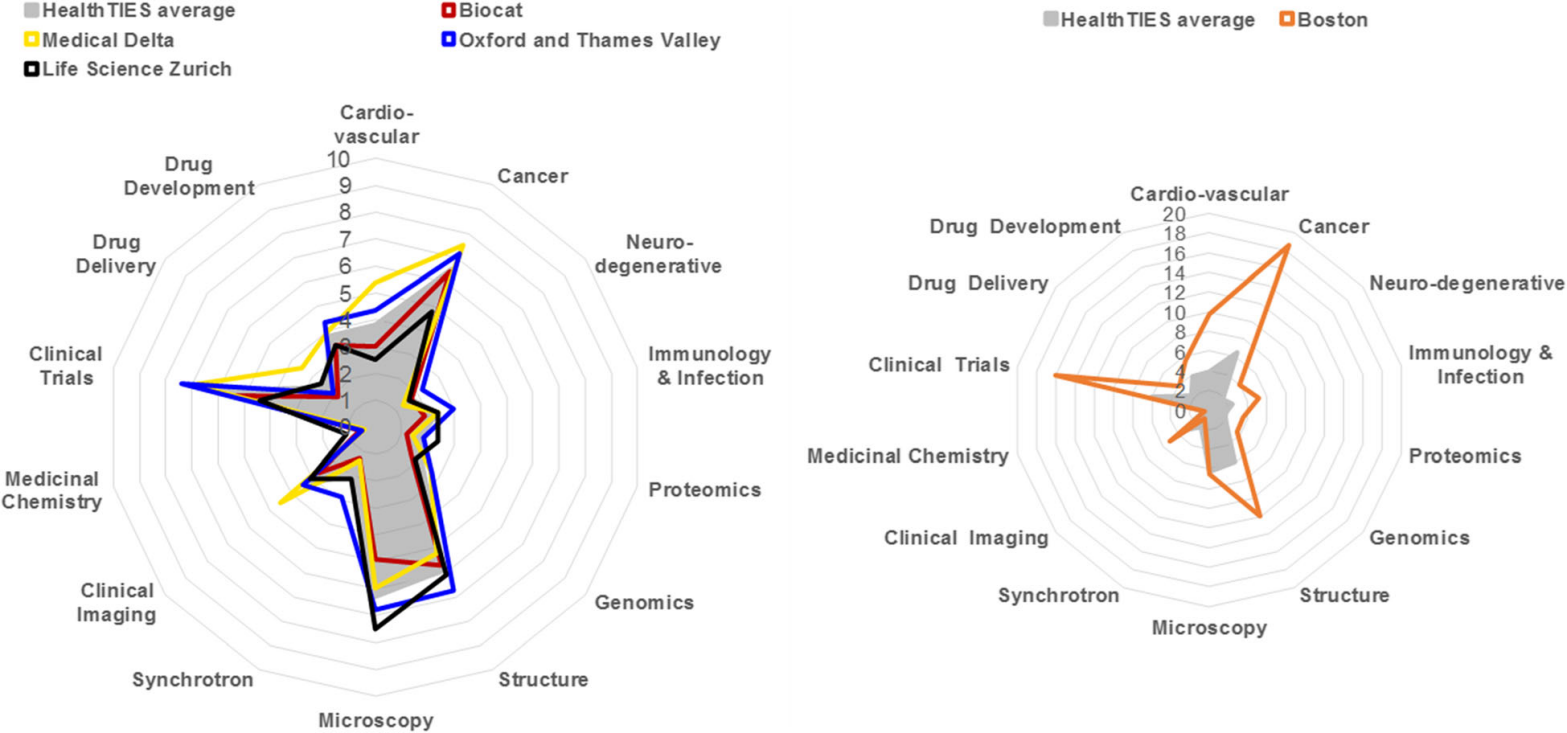
A comparative assessment of the knowledge and innovation capital using the HealthTIES H-index for disease and technology platforms, 2010. Data are expressed relative to a maximum of 7.5 in the best performing HealthTIES region

Selection of common bibliometric indicators enabled the comparison of the knowledge and innovation capital in regional clusters across operationally independent disease and technology platforms (Fig. 3). Strikingly, all four advanced regions had developed a relatively similar shape of disease and technology platform profiles, yet with distinctive strengths and weaknesses. For example, the strength of microscopy in Zürich and its weakness in Barcelona (Bioregion of Catalonia; BioCat), or the strength of clinical trials in Oxford and Medical Delta and their weakness in Zürich.

We also compared the average HealthTIES disease and technology platform profile with the profile of Boston, which is a renowned metropolitan area in life science innovation [52]. We found that the shape of disease and technology platform profiles was similar, but of significantly greater magnitude in Boston (Fig. 3). The relative similarity of regional profiles across Europe, especially with respect to cancer and cardiovascular disease, is likely to reflect the relatively similar priorities for healthcare funders. By contrast, the relatively limited activity in the public domain in chemistry and drug development is likely to reflect the concentration of this activity in the pharmaceutical industry.

#### Comparative assessment of the innovation cycle using the HealthTIES Innovation Index

**Fig. 4:**
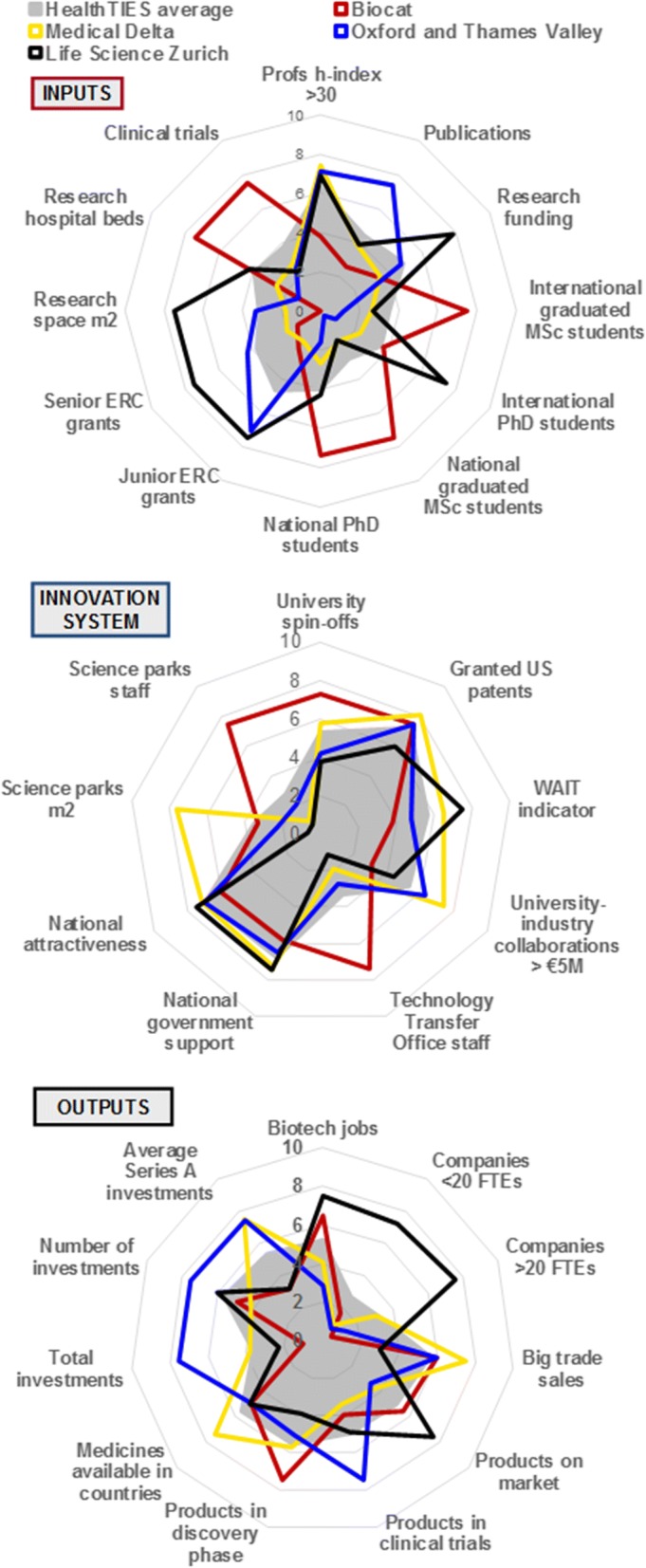
A comparative assessment of the innovation cycle using the HealthTIES Innovation index, 2010

The profiles obtained for each regional cluster in each of the three phases of the innovation ecosystem were surprisingly divergent (Fig. 4 and Additional file 1: Figure S1–S5). Thus, with a single time point or snapshot, it was difficult to determine the exact cause and effect relationships between indicators. This could also suggest that no one uniform template for regional innovation clusters exists in Europe, and that variation may be regional and context dependent.

In support of the utility of the HT Innovation index over time, the changes in data over two periods was determined in two regions where we could reassess all indicators (Additional file 1: Figure S6). These data strongly suggest that there is a time-dependent dynamic of the investment, innovation and outputs along the innovation cycle, illustrating the need for temporal assessments to evaluate fluctuations and (in)stability of the cluster. Over a given time, Life Science Zürich developed both investment and products on the market, whereas Oxford and Thames Valley and Medical Delta have achieved major increases in investment, but with little change in product or outputs in this period. Yet, Oxford and Thames Valley have a number of products in development, and this probably reflects an emphasis on clinical trials. Without a quantifiable HT Innovation index with temporal evaluation, there is a risk that these data could be misinterpreted. For example, the effect of increasing external investment in one particular area may not have impact on wealth creation. Likewise, increasing forward intellectual property without correlated development of investment may not translate to potential impacts on healthcare outcomes.

### HealthTIES indexes inform regional action

The indexes were then used to identify the relative strengths, weaknesses and opportunities in each cluster to benchmark regions, and provide a set of common innovation indicators that could be readily applied to any comparable cluster over time. For the gross differences between regions, the strengths, weaknesses, opportunities and threats (SWOT) and gap analyses were internally validated within the HealthTIES consortium. Moreover, as medical and life sciences represent a sizable part of the research and innovation system in each of the HealthTIES regions, comparisons of our findings for medical and life science clusters in the HealthTIES regions with the rankings of their respective countries and administrative regions across all sectors appear to indicate a degree of external prima facie validity of the HealthTIES indicators and indexes. In line with our findings that Life Science Zürich outperforms other HealthTIES regions on the greatest number of indicators, the European Innovation Scoreboard shows that Switzerland is the overall innovation leader in Europe, outperforming all EU member states [4]. Similar to the Regional Innovation Scoreboard (RIS) [5] ranking, the Oxford and Thames Valley region was a high innovator and BioCat and Medical Delta (only partial RIS data) were medium-high innovators. The HealthTIES indexes indicate that Oxford and Thames Valley performs slightly better than BioCat and Medical Delta. Both the RIS and HealthTIES study revealed that Debrecen is a low innovator.

Importantly, some of the differences between medical and life science clusters in the HealthTIES regions could be addressed in the local regions (Additional file 1: Figure S7). In an effort to create the ‘innovation space’ under the assumptions of the ‘triple helix’ model, the initial regional and joint actions following the adoption of the HealthTIES Innovation Indicators included the installation of formal ‘triple helix’ organisations (consisting of academia, industry and government), professionalisation of the academic TTOs, investing in entrepreneurship education, combined with incubator facilities and international entrepreneurs in residence programmes (Box 1). A set of joint actions could also lead to coupling and collaboration of some of Europe’s most innovative biotech regions such as combining business development between regions, public–private R&D and entrepreneurship education. Developing a more detailed regional roadmap for further actions would require a more integrated approach with ‘triple helix’ actors, comprehensive time-dependent data and prospective evaluation following each initiative.

### Best practice in bioincubation

Identifying and sharing best practice in bioincubation provides an illustrative case of regional actions following the adoption of the HealthTIES innovation indicators. A bioincubator is a physical environment with labs and offices where companies can start up, but also have access to experienced entrepreneurs, venture capital vehicles, skills training, mentoring and business services located in close proximity to major universities, research institutions, hospitals and life science companies. Most often, bioincubators are new ‘triple helix’ organisational actors that cut across the university–industry–government boundaries. In three HealthTIES regions (BioCat, Medical Delta and Life Science Zürich), bioincubators proved to be successful in stimulating innovation and economic growth by creating new high-tech companies and jobs, and therefore the Oxford regional team carried out site visits and in-depth interviews to study best practice in setting up and running bioincubators with a view to developing a bioincubator for Oxford and Thames Valley.

Major challenges in learning from international best practice concerned the transferability of findings to the intended setting. First, the functioning of bioincubators appeared to be dependent on laws, taxes and other country-specific institutions. Therefore, to increase the transferability of best practice to the United Kingdom context, in addition to five bioincubators in HealthTIES regions, we studied a further six bioincubators in England and Scotland. Second, the location and proximity of a bioincubator to major universities, research institutions, hospitals and life science companies emerged as the most frequently cited strength of the bioincubators studied. To capitalise on this strength, we studied in-depth BioPartner Centre Leiden, which emulates the proximity of the university and hospitals in Oxford better than any other bioincubator studied. Third, funding and sustainability in the first ten years of functioning emerged as the most frequently cited weakness of the bioincubators studied. There was a high degree of variation in how different bioincubators raised the investment necessary to build and equip the actual physical environment, attracted start-up companies, and generated revenue from multiple public and private sources over time. As no uniform business model emerged, we summarised a diverse range of best practice in setting up and running bioincubators in a business model canvas (Additional file 2).

## Discussion

This study reports the results and outcomes from the European FP7 Regions of Knowledge HealthTIES project. Specifically, our study provides tools for the measurement and benchmarking of university–industry–government innovation in selected European medical and life science clusters. The general outcome is that the HealthTIES indicators and indexes complement the existing generic tools for measuring innovation performance across all sectors at the level of countries and large administratively defined regions by focussing on actual innovation clusters in one specific sector. The very nature of this comparative process means that many indicators that are potentially informative were subsequently not used because of the lack of universal availability. There are therefore limitations to all such tools and indicators that are based on what can be measured, rather than on what should be measured. With respect to these caveats, the HealthTIES indicators and indexes have value, as they been co-developed and validated internally within the HealthTIES consortium of regional partners with representation from academia, government and industry, and appear to have a degree of external prima facie validity.

In addition, the HealthTIES indicators and indexes provided ample data for SWOT analysis, which in turn formed the basis of our regional and joint action plans. Our analysis shows that, although HealthTIES innovation regions possess a strong knowledge base and innovation infrastructure in the four mature clusters, and a strong potential for development in the mentoring cluster, they all significantly lag behind the world-leading Boston cluster (Fig. 3). This is a reflection of Europe being outperformed by the global innovation leaders such as South Korea, Canada, Australia, Japan and the United States [4]. Although the EU still has a clear innovation performance lead over many other countries globally, this lead is declining rapidly and comes under threat from countries such as China, whose innovation performance growth is three times that of the EU [4].

HealthTIES had regional value, as we used the results from the comparative assessment of the innovation cycle to identify and share best practice and inform regional and joint action plans to increase the competitiveness of the HealthTIES regions. There was also a synergistic effect from involving regional stakeholders across academia, industry and government in gathering and interpreting data. The HealthTIES project also raised awareness and increased commitment to change among the traditional ‘triple helix’ partners (academia, government, and medical and life science industry), new partners such as insurance companies and private investors, and the general public.

With regard to the particular areas of the knowledge and innovation capital, our study shows a major deficiency in the area of medical chemistry across all five regions. The lack of critical mass in this area in academia and publicly funded research institutes may be compensated by a larger volume of research in the pharmaceutical industry. All of the regions appear to be dependent on a steady drug development pipeline in the pharmaceutical industry, including vaccines and biological agents. In view of the changes that are ongoing in the pharmaceutical industry, these dependencies present a potential threat to the continued high-level activity in the HealthTIES clusters. For example, a major fall in revenue for pharma is expected as the patent lifetime of many drugs is coming to an end and they approach what has been termed the ‘patent cliff’ [53]. This fall in revenue and the increasing costs of getting drugs through Phase III, United States Food and Drug Administration and European Medicines Agency approval, mean that many drug development programmes are folding [54]. To counteract this threat in the context of P4 (predictive, preventive, personalised, participatory) medicine, academia and government across Europe may need to play a more active role in basic research and drug development in partnership with industry.

### Limitations and future research

The HealthTIES indicators and indexes have a number of limitations. First, our study was based on the assumptions of the ‘triple helix’ model to guide data collection, analysis and actions. Testing the underlying assumptions was beyond the scope of the study, as the ‘triple helix’ was regarded as a starting point. As a result, our analysis and proposed actions focused on the dynamic interactions within the ‘triple helix’ to generate a pragmatic, measurable and effective evaluation of the parameters influencing the innovation cycle. It is likely that this approach will have focused activity at the expense of other sectors. For example, taking into the account the apparent dependency of the HealthTIES regions on research and drug development in the pharmaceutical industry, incentivising R&D investment in the pharmaceutical industry through tax breaks could be either more or equally advantageous to some of the actions we considered. We intend to repeat data collection and HealthTIES innovation indicator analysis after implementation of the initial regional and joint actions. The next steps in evaluating the utility of the HealthTIES indicators and subsequent actions should include wider evaluation of the outcomes of the actions based on the assumptions of the ‘triple helix’ model, as well as comparison of their advantages and disadvantages with a range of possible alternative actions to accelerate innovation.

Second, quantifying and measuring innovation is inherently challenging because, by definition, it is something novel and the settings within which it occurs are multidimensional and in many ways unique. To ensure comparability and quantitative measurability, our approach uses a variety of indirect innovation indicators (e.g. research funding, publications, patents and investments) as well as quantitative direct innovation indicators (e.g. new products on the market, new medicines, big sales). An inherent limitation of such an approach is that it does not focus on the qualitative characteristics of the object of innovation, i.e. innovative products, services and technologies. Future research may explore alternative approaches focusing directly on the object of innovation to investigate whether the new products, services and technologies are incremental improvements or disruptive innovations, whether they have a potential to become blockbuster drugs or high-quality services, and whether they have occurred within or outside of the formal medical and life science R&D sector.

Third, within the inherent limitations of the current approach, there are considerable challenges in obtaining complete, reliable and comparable datasets, particularly for innovation system and output indicators. We were unable to include in our analysis a number of indicators, data for which were not available for all regions, or were not comparable or quantifiable. Particularly, there may be limitations to the reliability of some of the data collected for the HealthTIES regions. For example, companies complete their own entries in the BiotechGate database, and so their entries may vary in terms of quality, consistency and reporting periods. Moreover, the current version of the innovation indicators is based on absolute rather than relative measurements and thus does not take into consideration differences among HealthTIES regions with regards to the size and quality of various sociodemographic characteristics. Future research may usefully develop a methodology to account for such differences and to collect more complete, reliable and comparable data, for example, through comprehensive and cross-validated innovation surveys.

Fourth, external validation of the HealthTIES indicators and indexes was beyond the scope of the current project. Future research may usefully validate them in other settings using both quantitative and qualitative validation techniques. In particular, there may be a problem in using some of the readily available indicators across different settings. For example, the patients WAIT indicator typically includes the innovations and new drugs that have undergone market authorisation but are yet to undergo Phase IV clinical trials to determine their long-term effects on large populations, as well as health technology assessment to determine their cost effectiveness. Thus, some innovations and new drugs included in this indicator in some settings may have adverse long-term effects or may not be cost-effective. Our experience of conducting a workshop with a broad range of experts including, among others, front-line clinicians to build a consensus on the selection and feasibility of innovation indicators suggest that member validation and consensus research are likely to be effective means to gather expert opinion and build a consensus on the utility and feasibility of different indicators in the context of different regions.

Finally, our study did not explore qualitative differences between the clusters with regard to the workforce, business practices and laws, and how they support or prevent innovation. Yet, our previous research in one of the HealthTIES regions shows that innovation, entrepreneurship and collaboration culture varies between different healthcare and research organisations and may affect both health and economic outcomes [20, 55, 56]. Moreover, women remain under-represented in leadership and management positions in European academic health centres [57], which indicates that research systems may not support the best science or fail to address topics that benefit women and men equitably [58]. Future research may usefully explore qualitative differences between organisations, sectors and regions using theory-building research designs to draw generalisations beyond the current sample of medical and life science clusters.

## Conclusion

Important sectors of the economy, such as medical and life sciences, increasingly revolve around a ‘triple helix’ of university–industry–government relations at both national and regional levels. In light of growing interdependencies between previously arms-length actors, it is vital for research and innovation stakeholders and policy-makers to have access to tools for measuring, monitoring and comparing ‘triple helix’ dynamics in key sectors. The HealthTIES indicators and indexes provide useful practical tools for the measurement and benchmarking of university–industry–government innovation in European medical and life science clusters. They have been developed and validated internally within the HealthTIES consortium and display a degree of external prima facie validity. The results from the comparative assessment of the innovation cycle have been used to identify and share best practice and inform regional and joint action plans to strengthen the competitiveness of the HealthTIES regions. Potentially, the tools and accompanying analyses can be used beyond the HealthTIES consortium to inform other regional governments, researchers and, possibly, large companies searching for their next location, analyse and benchmark ‘triple helix’ dynamics within their own networks over time, and to develop integrated public–private and cross-regional research and innovation strategies in Europe and beyond.

Box 1 Examples of initial regional and joint actions following the adoption of the HealthTIES innovation indicators **• BioCat:** Improving the technology transfer offices (TTOs) transfer efficiency through roundtables with investors. Increase external cooperation nationally and internationally through partnerships. Improve clinical trial organisation, in particular proposals on how to improve clinical trial management and protocol turnaround.

**• Észak-Alföld:** Improve further public–private investment through Innova regional innovation engine. Student/researcher Mentoring Programme to enhance forward intellectual property and innovation outputs. Improve the attraction and retention of foreign students and leadership e.g. with new European Research Area strategic Chair.

**• Medical Delta:** Develop an innovation ‘Ambition’ programme with an integrated investment and TTO structure. Joint MD professorships and joint education programme across the cluster. Set up an investment fund to support pre-competitive innovation. Create large R&D facilities for public–private R&D ventures maximising utility of science parks. Study TTO best practice and propose a new TTO format drawing on best practice.

**• Oxford and Thames Valley:** Address a major deficiency in the University’s ‘spin-out’ activity by developing an Oxford incubator. Study best practice in biotech incubation, secure approval from all stakeholders, raise funding from public investment (Oxford City Deal) and gain a planning permission. In Autumn 2015, building started on the Oxford BioEscalator – a new incubation facility to grow small spin-off companies into mid-sized companies.

**• Life Science Zürich:** Address a major deficiency in translational research through a new ‘triple helix’ organisation for translational medicine. Creation of a ripple helix organisation and action agenda for Life Science Zürich is in progress. Consider a potential translational research centre and establish medical translational trials. Conduct a study on successful European Research Council laureate candidates, followed by a proposal on how to improve leadership recruitment. Establish an entrepreneur in residence programme.

**• Joint actions:** HealthTIES partners developed a successful bid to the European Institute for Innovation and Technology (EIT) for a Knowledge and Innovation Community in Health. Participate in joint education in innovation programmes using the EIT Health STELLAR approach (Spark, Transform, Embrace, Lead, Leap, Amplify, Reward). Connect incubators as soft-landing places for exchange scientists, entrepreneurs and students (EIT Health).

## Additional files

Additional file 1: **Box S1.** Search protocol for HealthTIES (HT) H-indexes for disease and technology platforms, Web of Science. **Table S1.** Keywords used in the HT H-index analysis. **Figure S1.** Radar plots of the HT Innovation index for Input, Innovation System and Output Indicators (with weighted scores) for each region separately: Biocat (The HealthTIES average is shown in grey). **Figure S2.** Radar plots of the HT Innovation index for Input, Innovation System and Output Indicators (with weighted scores) for each region separately: Észak-Alföld (The HealthTIES average is shown in grey). **Figure S3.** Radar plots of the HT Innovation index for Input, Innovation System and Output Indicators (with weighted scores) for each region separately: Medical Delta (The HealthTIES average is shown in grey). **Figure S4.** Radar plots of the HT Innovation index for Input, Innovation System and Output Indicators (with weighted scores) for each region separately: Oxford and Thames Valley (The HealthTIES average is shown in grey). **Figure S5.** Radar plots of the HT Innovation index for Input, Innovation System and Output Indicators (with weighted scores) for each region separately: Life Science Zurich (The HealthTIES average is shown in grey). **Figure S6.** Clustered column diagrams of the HT Innovation index over time, 2010 and 2012. **Figure S7.** Strengths, weaknesses, opportunities and threats (SWOT) analyses for each region. (PDF 1712 kb)

Additional file 2: A business model canvas for bioincubators based on best practice in 11 bioincubators in Catalonia, South Holland, Zürich, England and Scotland, 2013. (PDF 192 kb)

## Abbreviations

BioCat: Bioregion of Catalonia
ERC: European Research Council
EU: European Union
FTE: full-time equivalent
HealthTIES: Healthcare, Technology and Innovation for Economic Success
HT H-index: HealthTIES h-index
R&D: research and development
RIS: Regional Innovation Scoreboard
STI: science, technology and innovation
SWOT: strengths, weaknesses, opportunities and threats
TTO: technology transfer office
WAIT: patients Waiting for Access to Innovative Treatment
