# Supplementary material for: New indicators and indexes for benchmarking university–industry–government innovation in medical and life science clusters: results from the European FP7 Regions of Knowledge HealthTIES project
Source: Health Res Policy Syst. 2019 Jan 28;17:10. doi: 10.1186/s12961-019-0414-5 (PMC6350323; doi:10.1186/s12961-019-0414-5)

Additional file 1

Additional File Box 1: Search protocol for HealthTIES H-indexes for disease and technology platforms, Web of Science

- Addresses: “Zurich”, “Barcelona” and “Debrecen” were used as search terms. Oxford was searched using “Univ Oxford”. Medical Delta results were derived from separate searches for “Leiden”, “Rotterdam” and “Delft” which were then combined in search histories using OR as the Boolean operator to eliminate duplications.

- Topics: terms as shown in Additional File Table 1, were searched individually for each location with the exception of “immunology” and “infectious disease” which were searched separately and then combined in search histories (also using OR). “Structure” was restricted using NOT to exclude “astronomy” and “astrophysics”.

- Timespan: years were set to 2001 to 2010 for all searches with the exception of “cancer” which was set from 2005 to 2010 due to the large number of articles in this field. The repeated measures two years later were set from 2001 to 2012 and 2005 to 2012 respectively.

- Citation report: once each search or combined searches had generated results, the h-index was retrieved by clicking on “Create Citation Report".

Additional File Table 1. Keywords used in the HealthTIES index (HT H-index) analysis

**Disease field or technology platform**	**Keyword**
Cardiovascular disease	Cardiovascular
Cancer	Cancer
Neurodegenerative disease	Neurodegenerative
Immunology and infectious disease	Immunology
	Infectious disease
Molecular Technology	Proteomics
	Genomics
	Structure
Imaging	Microscopy
	Synchrotron
	Clinical imaging
Drug design, development and delivery	Medicinal chemistry
	Clinical trials
	Drug delivery
	Drug development

Additional File Fig. 1 Radar plots of the HT Innovation index for Input, Innovation System and Output Indicators (with weighted scores) for each region separately: Biocat (The HealthTIES average is shown in gray).

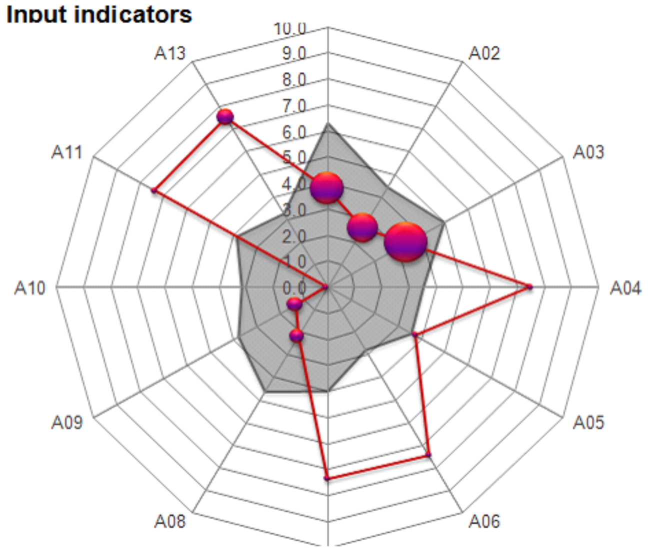

A01 Profs h-index >30; A02 Publications; A03 Research spending/funding; A04 International graduated MSc students; A05 International PhD students; A06 National graduated MSc students; A07 National PhD students; A08 Junior ERC grants; A09 Senior ERC grants; A10 Research space m^2; A11 Research hospital beds; A12 Clinical trials

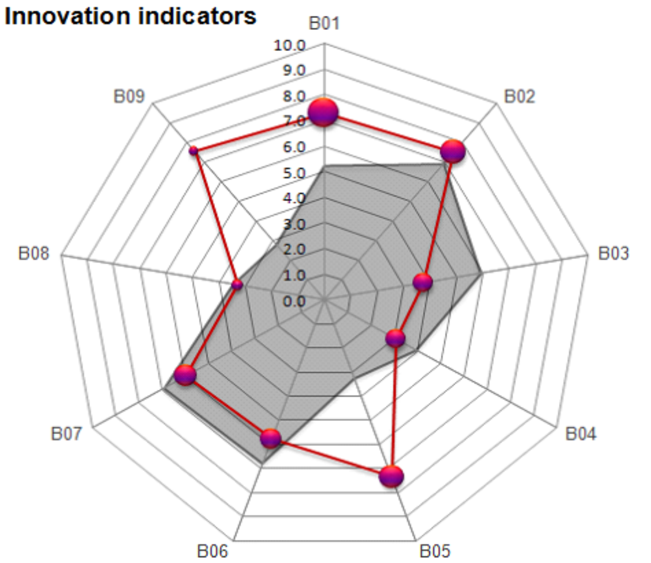

B01 Spin outs; B02 Granted US patents; B03 W.A.I.T. indicator; B04 Joint research projects ; B05 TTOs FTEs; B06 Governmental innovation support; B07 Regional attractiveness WEF GCR 2010-2011; B08 Science parks m^2; B09 Science parks FTEs

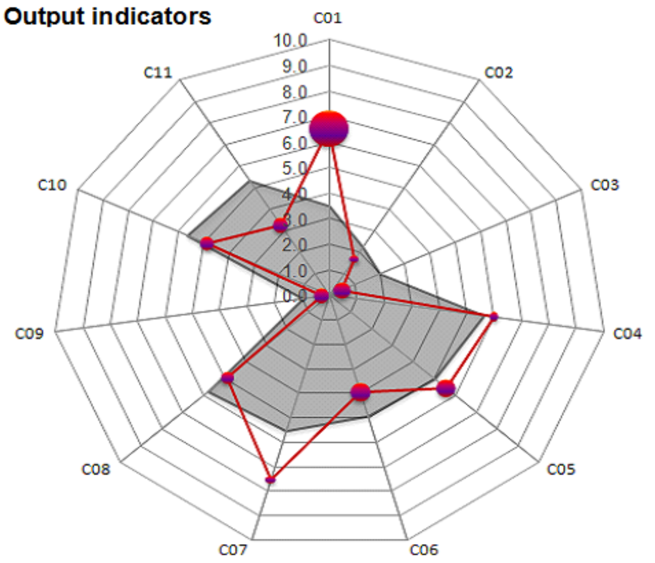

C01 FTEs working in HT disciplines; C02 Companies <20; C03 Companies >20; C04 Big trade sales; C05 Products on market; C06 Products Phase I-III + NDA; C07 Products in discovery phase; C08 Medicines available in countries; C09 Total investments; C10 # investments; C11 Average Series A investments

Additional File Fig. 2 Radar plots of the HT Innovation index for Input, Innovation System and Output Indicators (with weighted scores) for each region separately: Észak-Alföld (The HealthTIES average is shown in gray).

Input indicators

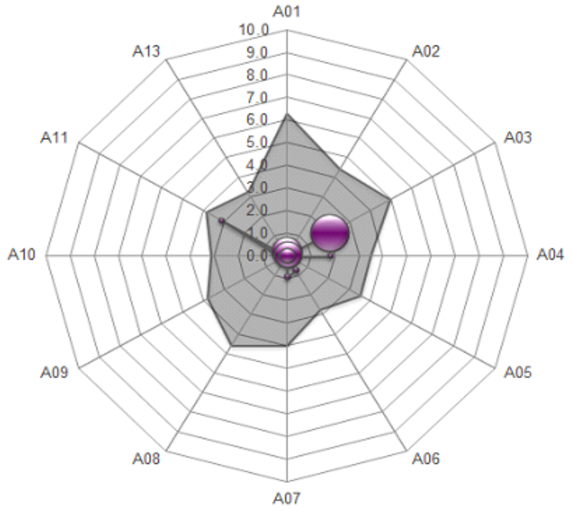

A01 Profs h-index >30; A02 Publications; A03 Research spending/funding; A04 International graduated MSc students; A05 International PhD students; A06 National graduated MSc students; A07 National PhD students; A08 Junior ERC grants; A09 Senior ERC grants; A10 Research space m^2; A11 Research hospital beds; A12 Clinical trials

Innovation indicators

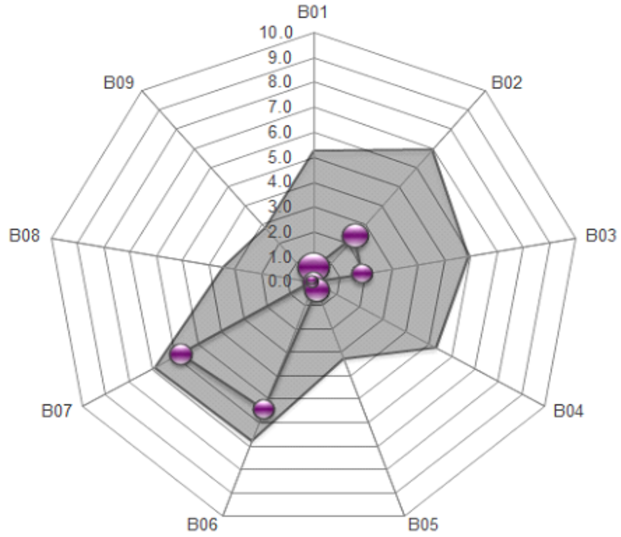

B01 Spin outs; B02 Granted US patents; B03 W.A.I.T. indicator; B04 Joint research projects ; B05 TTOs FTEs; B06 Governmental innovation support; B07 Regional attractiveness WEF GCR 2010-2011; B08 Science parks m^2; B09 Science parks FTEs

Output indicators

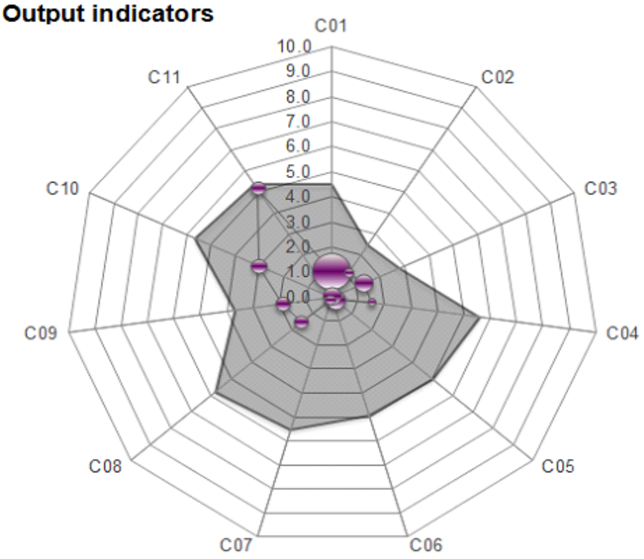

C01 FTEs working in HT disciplines; C02 Companies <20; C03 Companies >20; C04 Big trade sales; C05 Products on market; C06 Products Phase I-III + NDA; C07 Products in discovery phase; C08 Medicines available in countries; C09 Total investments; C10 # investments; C11 Average Series A investments

Additional File Fig. 3 Radar plots of the HT Innovation index for Input, Innovation System and Output Indicators (with weighted scores) for each region separately: Medical Delta (The HealthTIES average is shown in gray).

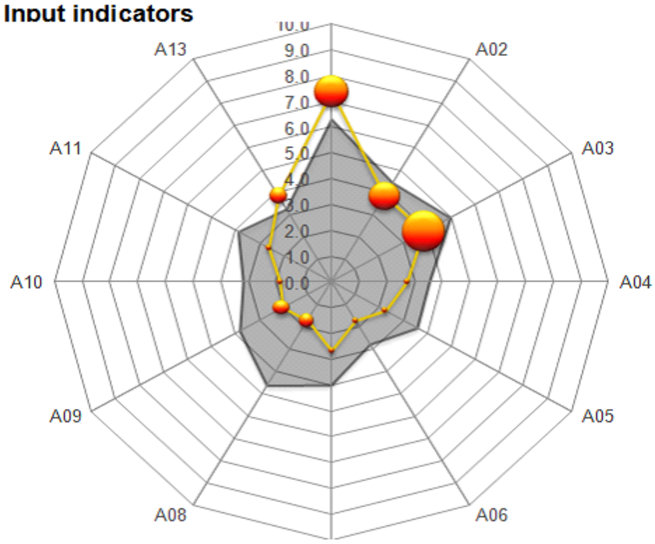

A01 Profs h-index >30; A02 Publications; A03 Research spending/funding; A04 International graduated MSc students; A05 International PhD students; A06 National graduated MSc students; A07 National PhD students; A08 Junior ERC grants; A09 Senior ERC grants; A10 Research space m^2; A11 Research hospital beds; A12 Clinical trials

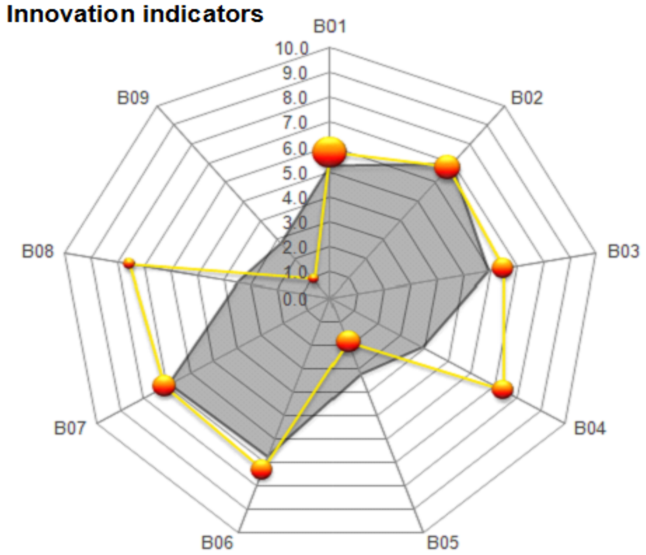

B01 Spin outs; B02 Granted US patents; B03 W.A.I.T. indicator; B04 Joint research projects ; B05 TTOs FTEs; B06 Governmental innovation support; B07 Regional attractiveness WEF GCR 2010-2011; B08 Science parks m^2; B09 Science parks FTEs

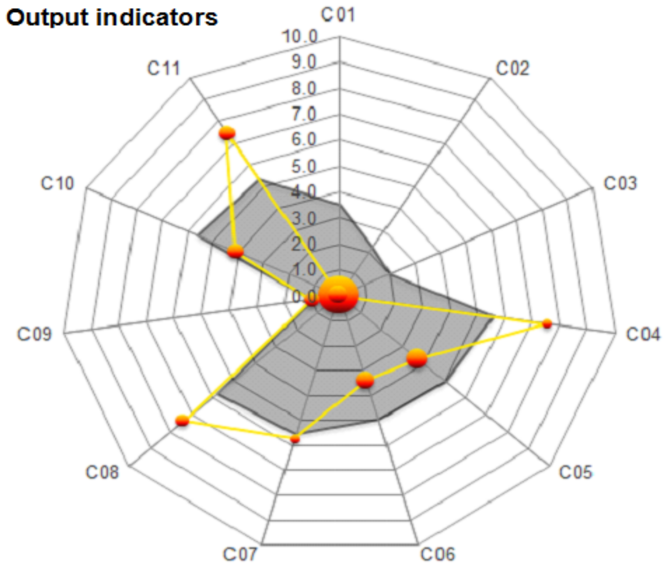

C01 FTEs working in HT disciplines; C02 Companies <20; C03 Companies >20; C04 Big trade sales; C05 Products on market; C06 Products Phase I-III + NDA; C07 Products in discovery phase; C08 Medicines available in countries; C09 Total investments; C10 # investments; C11 Average Series A investments

Additional File Fig. 4 Radar plots of the HT Innovation index for Input, Innovation System and Output Indicators (with weighted scores) for each region separately: Oxford and Thames Valley (The HealthTIES average is shown in gray).

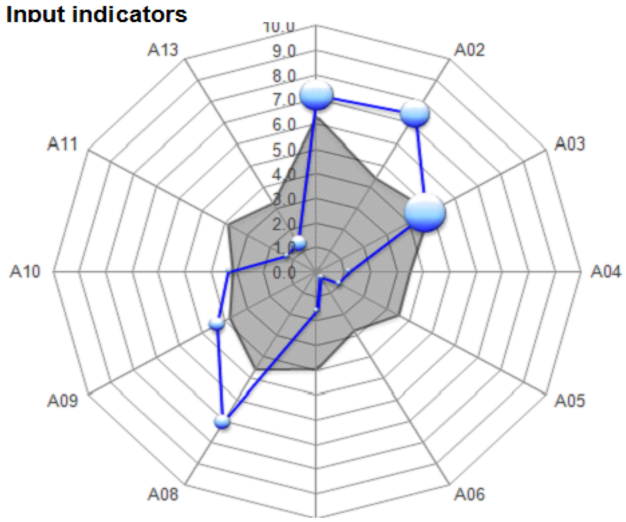

A01 Profs h-index >30; A02 Publications; A03 Research spending/funding; A04 International graduated MSc students; A05 International PhD students; A06 National graduated MSc students; A07 National PhD students; A08 Junior ERC grants; A09 Senior ERC grants; A10 Research space m^2; A11 Research hospital beds; A12 Clinical trials

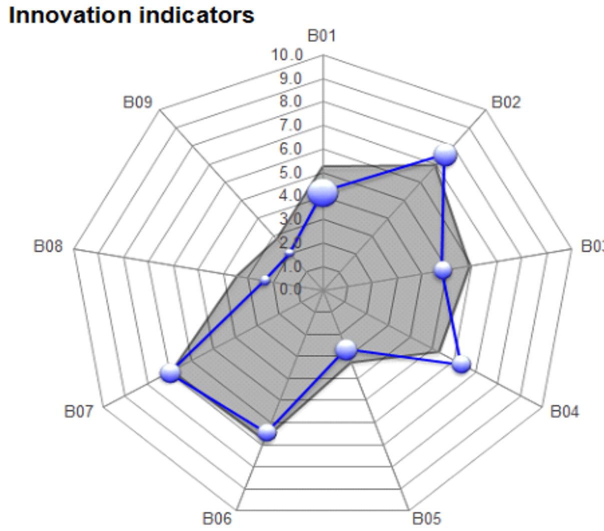

B01 Spin outs; B02 Granted US patents; B03 W.A.I.T. indicator; B04 Joint research projects ; B05 TTOs FTEs; B06 Governmental innovation support; B07 Regional attractiveness WEF GCR 2010-2011; B08 Science parks m^2; B09 Science parks FTEs

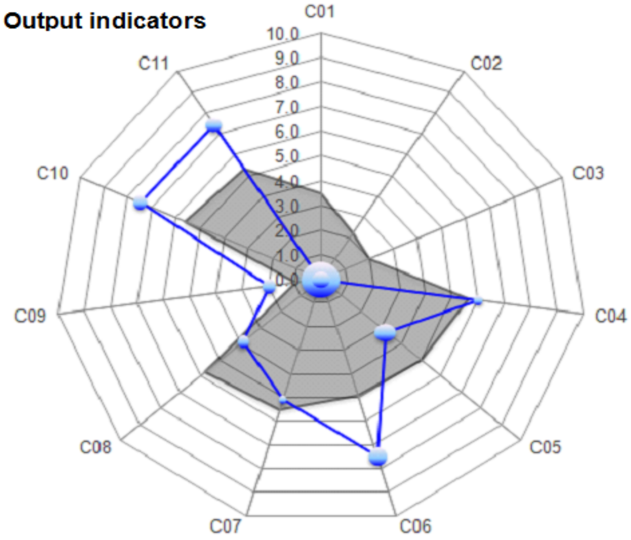

C01 FTEs working in HT disciplines; C02 Companies <20; C03 Companies >20; C04 Big trade sales; C05 Products on market; C06 Products Phase I-III + NDA; C07 Products in discovery phase; C08 Medicines available in countries; C09 Total investments; C10 # investments; C11 Average Series A investments

Additional File Fig. 5 Radar plots of the HT Innovation index for Input, Innovation System and Output Indicators (with weighted scores) for each region separately: Life Science Zurich (The HealthTIES average is shown in gray.

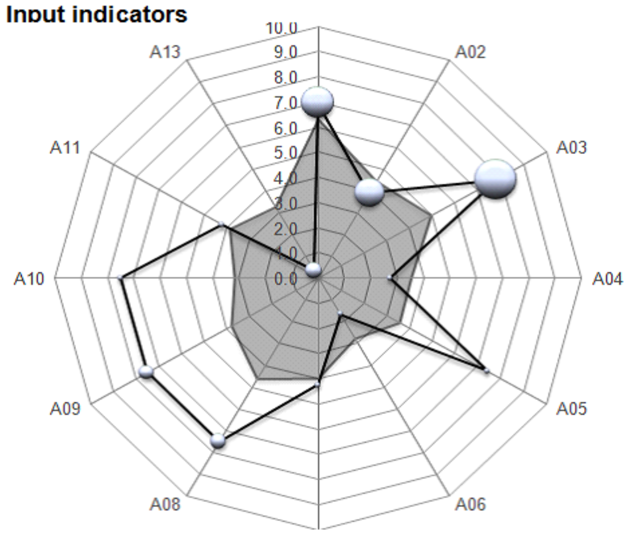

A01 Profs h-index >30; A02 Publications; A03 Research spending/funding; A04 International graduated MSc students; A05 International PhD students; A06 National graduated MSc students; A07 National PhD students; A08 Junior ERC grants; A09 Senior ERC grants; A10 Research space m^2; A11 Research hospital beds; A12 Clinical trials

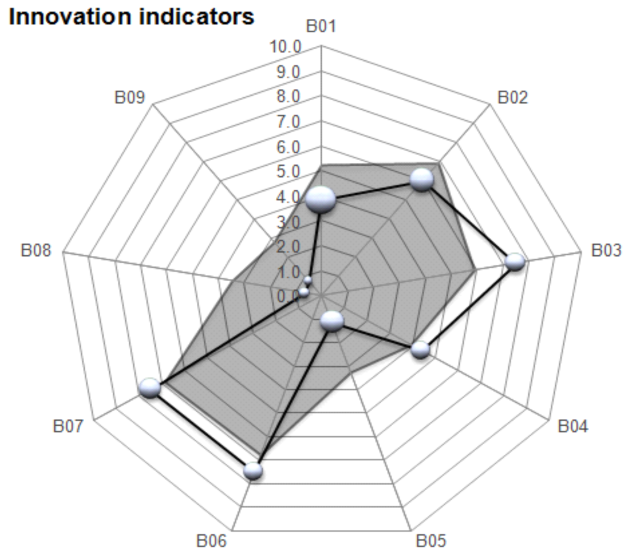

B01 Spin outs; B02 Granted US patents; B03 W.A.I.T. indicator; B04 Joint research projects ; B05 TTOs FTEs; B06 Governmental innovation support; B07 Regional attractiveness WEF GCR 2010-2011; B08 Science parks m^2; B09 Science parks FTEs

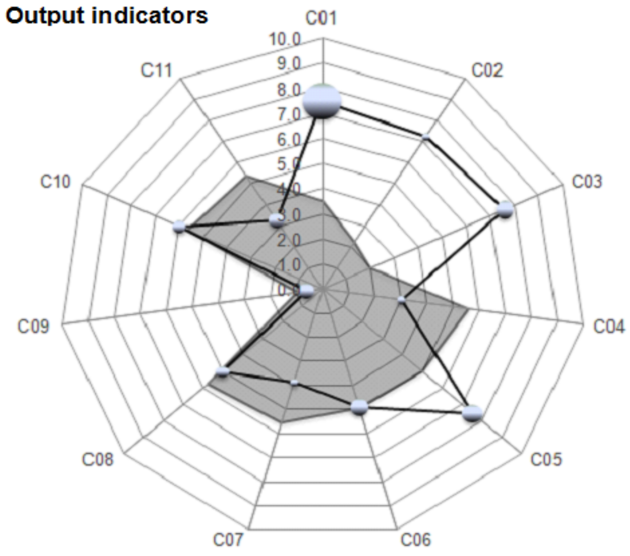

C01 FTEs working in HT disciplines; C02 Companies <20; C03 Companies >20; C04 Big trade sales; C05 Products on market; C06 Products Phase I-III + NDA; C07 Products in discovery phase; C08 Medicines available in countries; C09 Total investments; C10 # investments; C11 Average Series A investments

Additional File Fig. 6 Clustered column diagrams of the HT Innovation index over time, 2010 and 2012.

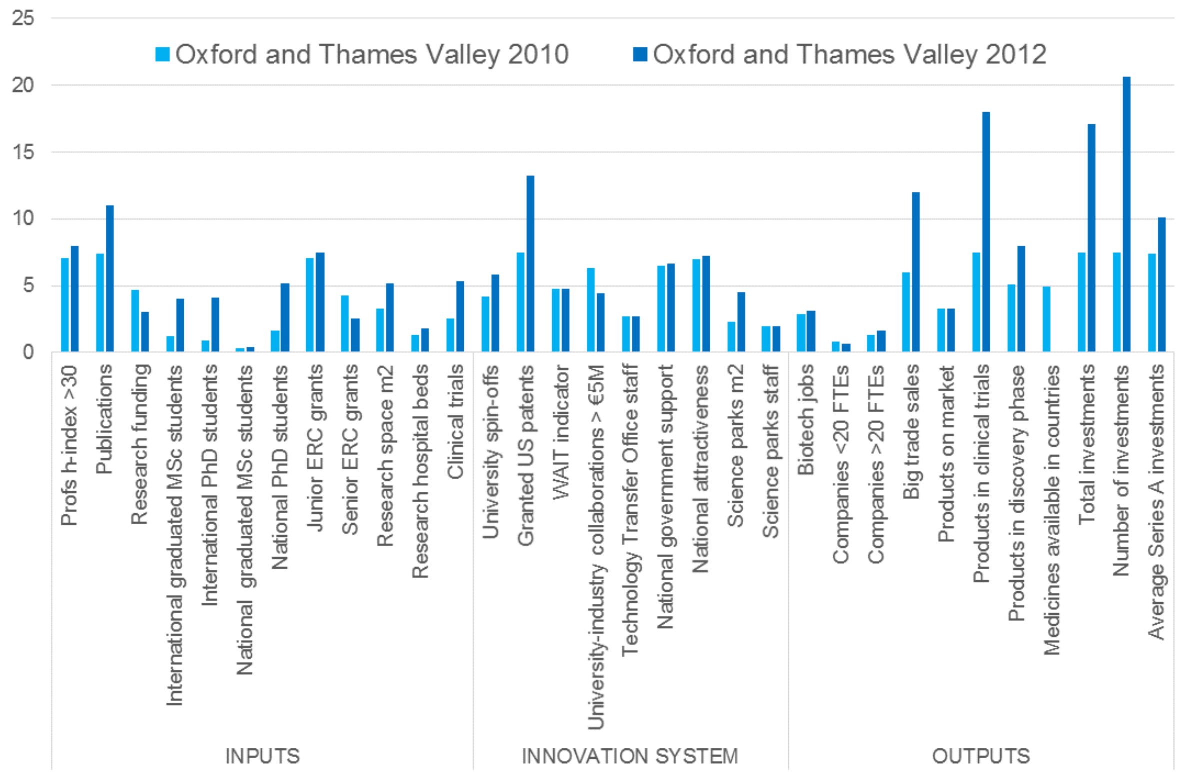

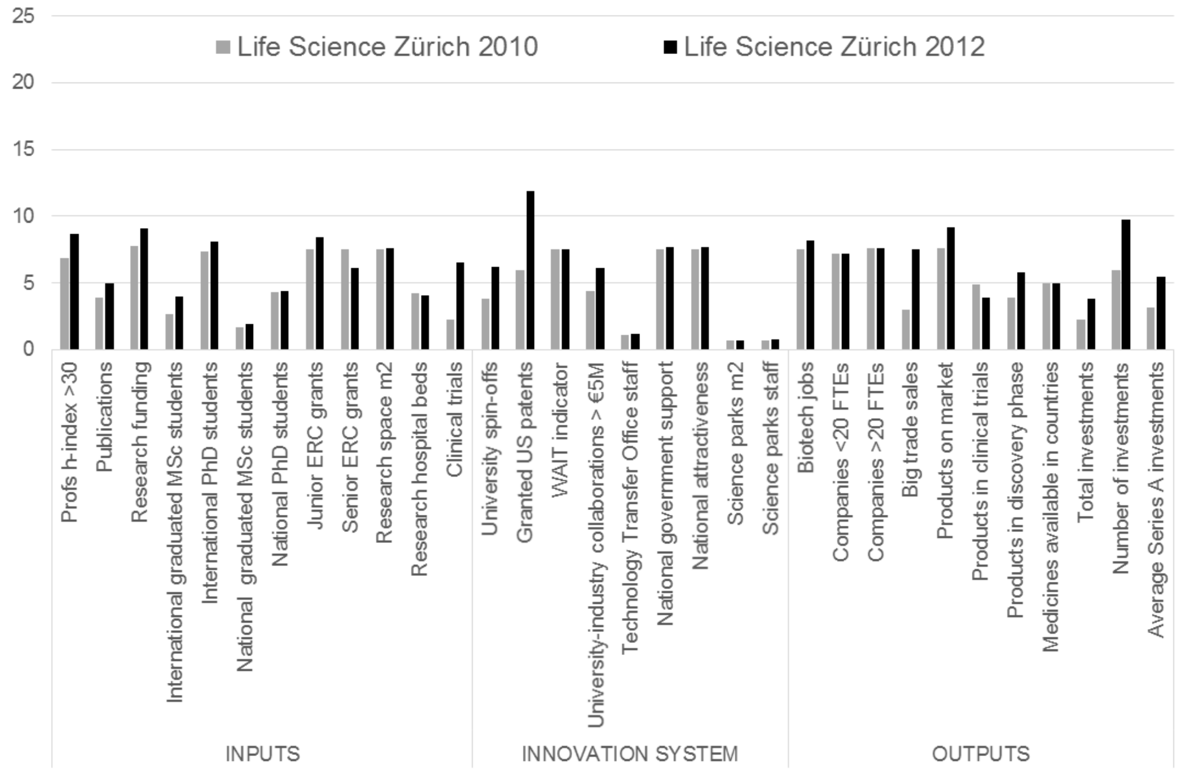

Additional File Fig. 7 Strengths, weaknesses, opportunities and threats (SWOT) analyses for each region.

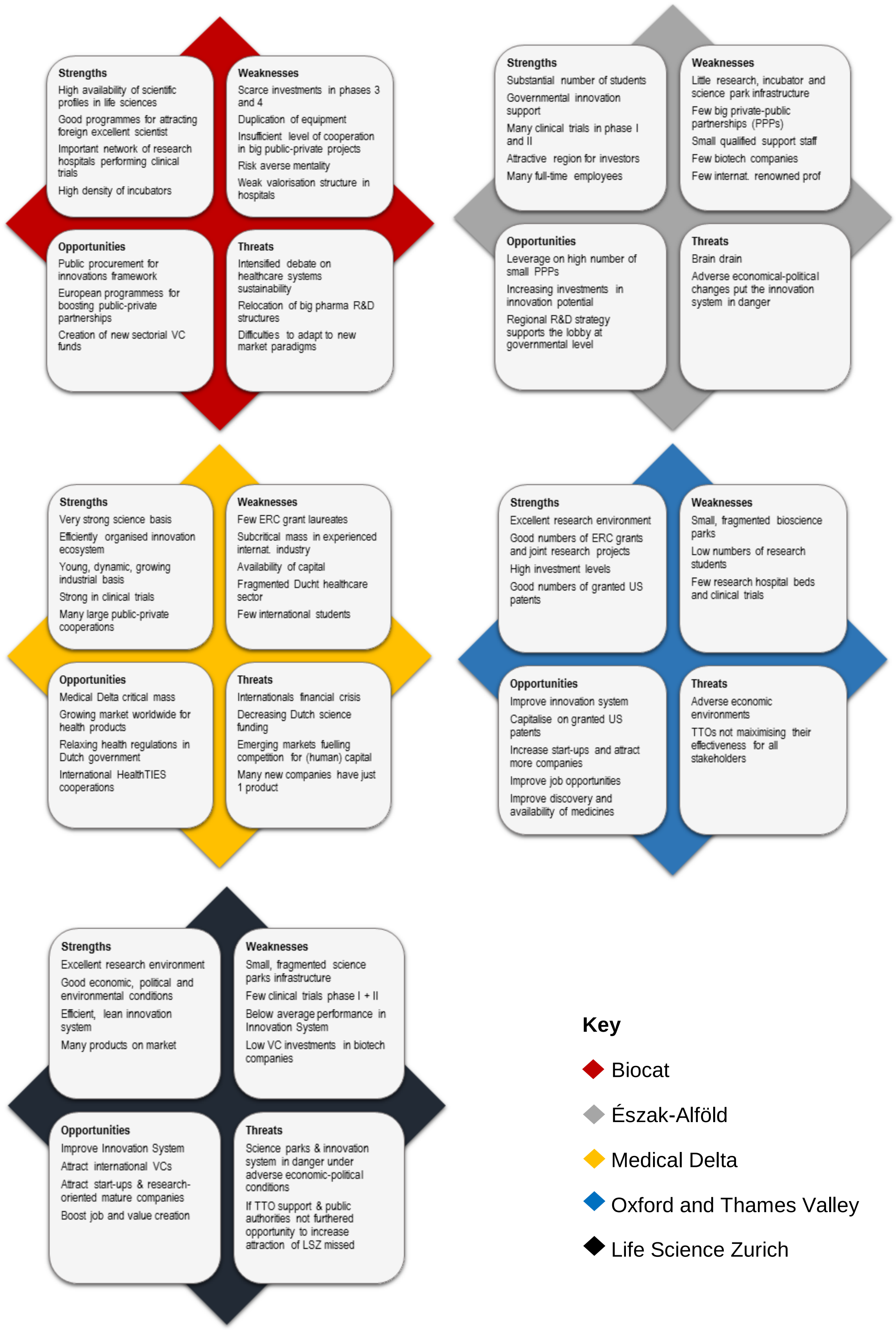

Supplement: Supplementary file 1 — Box S1. Search protocol for HealthTIES (HT) H-indexes for disease and technology platforms, Web of Science. Table S1. Keywords used in the HT H-index analysis. Figure S1. Radar plots of the HT Innovation index for Input, Innovation System and Output Indicators (with weighted scores) for each region separately: Biocat (The HealthTIES average is shown in grey). Figure S2. Radar plots of the HT Innovation index for Input, Innovation System and Output Indicators (with weighted scores) for each region separately: Észak-Alföld (The HealthTIES average is shown in grey). Figure S3. Radar plots of the HT Innovation index for Input, Innovation System and Output Indicators (with weighted scores) for each region separately: Medical Delta (The HealthTIES average is shown in grey). Figure S4. Radar plots of the HT Innovation index for Input, Innovation System and Output Indicators (with weighted scores) for each region separately: Oxford and Thames Valley (The HealthTIES average is shown in grey). Figure S5. Radar plots of the HT Innovation index for Input, Innovation System and Output Indicators (with weighted scores) for each region separately: Life Science Zurich (The HealthTIES average is shown in grey). Figure S6. Clustered column diagrams of the HT Innovation index over time, 2010 and 2012. Figure S7. Strengths, weaknesses, opportunities and threats (SWOT) analyses for each region. (PDF 1712 kb) [file 12961_2019_414_MOESM1_ESM.pdf]
