# Supplementary material for: New indicators and indexes for benchmarking university–industry–government innovation in medical and life science clusters: results from the European FP7 Regions of Knowledge HealthTIES project
Source: Health Res Policy Syst. 2019 Jan 28;17:10. doi: 10.1186/s12961-019-0414-5 (PMC6350323; doi:10.1186/s12961-019-0414-5)

Additional file 2

A business model canvas for bioincubators based on best practice in eleven bioincubators in Catalonia, South Holland, Zürich, England, and Scotland, 2013.

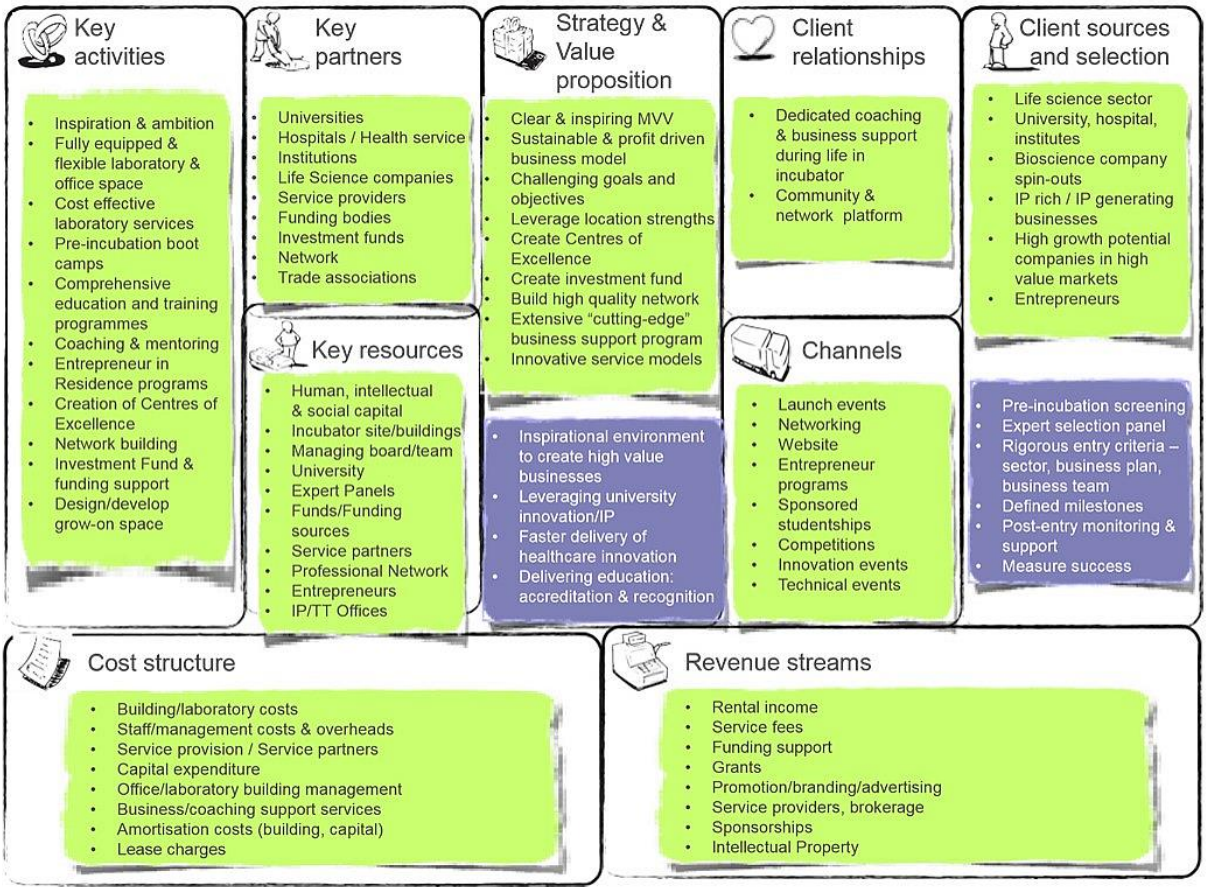

Supplement: Supplementary file 2 — A business model canvas for bioincubators based on best practice in 11 bioincubators in Catalonia, South Holland, Zürich, England and Scotland, 2013. (PDF 192 kb) [file 12961_2019_414_MOESM2_ESM.pdf]
